# Study on the Process Characteristics Based on Joule Heat of Sliding-Pressure Additive Manufacturing

**DOI:** 10.3390/ma16052017

**Published:** 2023-02-28

**Authors:** Kaiyue Ma, Suli Li, Chao Xu, Zhuang Gao, Laixia Yang, Bingheng Lu

**Affiliations:** 1 School of Mechanical Engineering, Xi’an University of Science and Technology, Xi’an 710054, China; 2 National Innovation Institute of Additive Manufacturing, Xi’an 710300, China; 3 State Key Laboratory for Manufacturing Systems Engineering, Xi’an Jiaotong University, Xi’an 710049, China

**Keywords:** additive manufacturing, Joule heat, process parameters, aspect ratio, dilution ratio

## Abstract

This study developed an experimental system based on Joule heat of sliding-pressure additive manufacturing (SP-JHAM), and Joule heat was used for the first time to accomplish high-quality single-layer printing. The roller wire substrate is short-circuited, and Joule heat is generated to melt the wire when the current passes through. Through the self-lapping experimental platform, single-factor experiments were designed to study the effects of power supply current, electrode pressure, contact length on the surface morphology and cross-section geometric characteristics of the single-pass printing layer. Through the Taguchi method, the effect of various factors was analyzed, the optimal process parameters were obtained, and the quality was detected. The results show that with the current increase, the aspect ratio and dilution rate of a printing layer increase within a given range of process parameters. In addition, with the increase in pressure and contact length, the aspect ratio and dilution ratio decrease. Pressure has the greatest effect on the aspect ratio and dilution ratio, followed by current and contact length. When a current of 260 A, a pressure of 0.60 N and a contact length of 1.3 mm are applied, a single track with a good appearance, whose surface roughness Ra is 3.896 μm, can be printed. Additionally, the wire and the substrate are completely metallurgically bonded with this condition. There are also no defects such as air holes and cracks. This study verified the feasibility of SP-JHAM as a new additive manufacturing strategy with high quality and low cost, and provided a reference for developing additive manufacturing technology based on Joule heat.

## 1. Introduction

In recent years, metal additive manufacturing has gradually become one of the key research directions in advanced manufacturing. Rapid prototyping, manufacturing of complex design structures, direct molding of parts without mold, surface strengthening, and repairing materials are some advantages of additive manufacturing (AM) technologies over conventional tooling-based methods such as casting or machining [1,2,3]. Its applications have expanded in numerous areas, including the medical, aerospace, automotive, construction, defense, and consumable sectors [4]. Moreover, in high-tech fields such as advanced equipment, unmanned aerial vehicles and artificial satellites, there is huge market demand for micro-components and structures with the characteristic size of 10~100 mm, because of the trend of low energy consumption and miniaturization. Therefore, various processing and forming manufacturing methods based on micro-components have become research hotspots [5]. At present, mainstream high-energy beam fuse additive manufacturing technologies are represented by laser metal deposition (LMD) [6,7], electron beam additive manufacturing (EBAM) [8,9], wire arc additive manufacturing (WAAM) [10], etc. LMD and EBAM have the merits of high precision and flexibility, but expensive laser and electron guns greatly increase the printing cost. LMD is not suitable for printing metals with high surface reflectivity, such as aluminum and its alloys [11,12,13,14,15]. WAAM reduces the printing cost and improves the printing efficiency; however, more post-processing is required because of the greater roughness [16,17,18,19].

Improving the forming quality and reducing the forming cost of additive manufacturing has gradually attracted the industry’s attention [20,21]. Some scholars [22,23] have used the hot-wire process combined with other heat sources to print, which effectively improves the forming quality. The principle of the hot-wire process is to preheat the wire using a separate power supply and then send it into the molten pool. This process benefits the metallurgical control, energy utilization rate and deposition rate [24]. Shinozaki et al. [25] carried out an experimental study on the weld formation of hot-wire laser welding process parameters. The results show that the current in the molten pool is suitable for producing welding wire melt; the weld is denser, and the microstructure will be more uniform. Wonthaisong et al. [26] found that a lower dilution rate and higher hardness of welding metal can be accomplished using hot-wire technology. Pattarawadee et al. [3] used the hot-wire plasma welding process to inspect the printed thin-walled wall. They found that the combination of the hot-wire and welding process seemed to have the potential to achieve high efficiency and faster welding, and the samples had better mechanical properties. However, the above studies all require the compound heating of two heat sources, which increases the complexity of equipment and technology at a high cost.

To meet the market demand for low-cost and small components’ manufacturing, this research group put forward an additive manufacturing technology based on Joule’s heat principles to melt the wires. Using this technology, processing accuracy is guaranteed by the Joule heat generated by the current flowing through the wires. Besides, the forming quality is improved through a small melting pool, micro-metallurgy, and rapid solidification technology. At the same time, power waste and equipment costs can be reduced due to the closed-loop energy transfer and simple equipment structure. In a previous study, an experimental system based on Joule heat of molten-droplet additive manufacturing (MD-JHAM) was developed by this research group [27]. By short-circuiting the wire substrate, the maximum Joule heat was generated at the contact position between the wire and the substrate. After the tip of the wire was heated, the droplet was formed by surface tension and gravity and then transferred to the substrate. Therefore, continuous forming was realized with the movement of the numerical control platform, which proved the feasibility of this technology. Although low-cost multi-layer printing can be accomplished by MD-JHAM, the forming quality needs to be improved.

In this study, based on the Joule heat principle, an experimental system SP-JHAM was developed. The law and degree of effects of power supply current, electrode pressure, contact length on the forming appearance and cross-section geometry of a single printed layer were studied by single-factor experiment and orthogonal experiment. A set of optimal process parameters were optimized by the Taguchi method. The surface roughness and internal quality of a single-pass printing layer were tested and analyzed, which verified the feasibility of this process. This study aims to guide the application and popularization of the additive manufacturing technology of Joule heat, whilst to promoting the development of additive manufacturing technology of small components with high quality and low cost.

## 2. Materials and Methods

### 2.1. Materials

The stainless steels 304 and 316 L are widely used in automobiles, metallurgy, petrochemical, construction, machinery, and other fields because of their good corrosion resistance, high tensile strength, impact resistance, good ductility and wear resistance [28].

In this study, 304 stainless steel wire with diameter of 0.3 mm is taken as the research object. The substrate is 316 L stainless steel whose specification is 100 mm × 100 mm × 5 mm. Both the wire and substrates were purchased from TISCO, Taiyuan, China. The chemical compositions of these two materials are shown in Table 1, and the physical properties of these two materials at room temperature (20 °C) are shown in Table 2 [29,30].

### 2.2. Equipment

**Figure 1:**
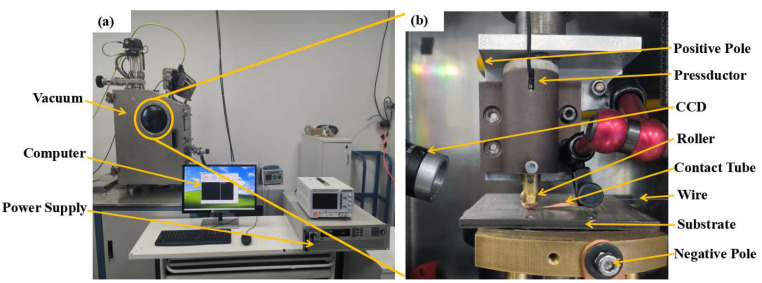
Equipment of SP-JHAM experimental system: (**a**) Overview of SP-JHAM experimental system, (**b**) Platform of SP-JHAM experimental system inside the vacuum.

The equipment of the SP-JHAM experimental system is shown in Figure 1. The positive pole of a programmable power supply IT6010D-80-300 (ITECH, China, Nanjing) was connected to the roller, while the negative pole of the power supply was connected to the metal substrate. The copper roller contacted the wire as a downward movement of the computer numerical control (CNC) machine tool CNC-M1 (XIANHE, Zibo, China). As a result, the current through the positive pole of the power supply, copper roller, metal wire and substrate to the negative pole of the power supply formed a loop. The maximum Joule heat was generated in the contact position of the roller and the wire, resulting in the local melting of the wire. Therefore, the wire and the substrate were welded together. As the platform moved along the horizontal direction, the wire was pulled in with the specified speed. Finally, the additive manufacturing of quantitative melting of the wire at the roller contact position was realized.

In the experiment, the pressure was controlled by the *Z*-axis movement of the platform, whose value was read by a pressure sensor FA402B (FIBOS, Changzhou, China) with an accuracy of 0.01 N. The printing process was recorded by a charge-coupled device (CCD) high-speed camera. To prevent oxidation and ensure the stability of the printing process, the experiment was carried out in a customized vacuum box (SHUAISHI, Xi’an China). After the vacuum box was closed, a double-stage pump TRIVAC D60T (LEYBOLD, Cologne, Germany) pumped atmospheric pressure in the vacuum box below 1 Pa and then started a semi-magnetic molecular pump TURBOVAC 90i (LEYBOLD, Cologne, Germany) to pump atmospheric pressure in the vacuum box to 7 × 10^−2^ Pa and keep it below 7 × 10^−2^ Pa. A high-precision vacuometer ITR90N (LEYBOLD, Cologne, Germany) was used to ensure the accuracy of the vacuum degree in the vacuum box. Clamps and fixtures firmly clamped the base plate to prevent it from moving or deflecting during welding.

The output current of the power supply passes through the copper roller, and the metal wire and the substrate generate Joule heat (*Q*) [31], which is obtained by Joule’s law:

(1) $$Q=\int_{0}^{t_{1}}i^{2}\left(t\right)Rdt$$

where *i* is the instantaneous current in the electrifying stage, *t*_1_ is the electrifying time of the metal wire, and *r* is the total resistance in the electrifying process.

It can be seen that Joule heat *Q* has a quadratic functional relationship with current *i* and a linear relationship with current *i* and resistance *R*.

**Figure 2:**
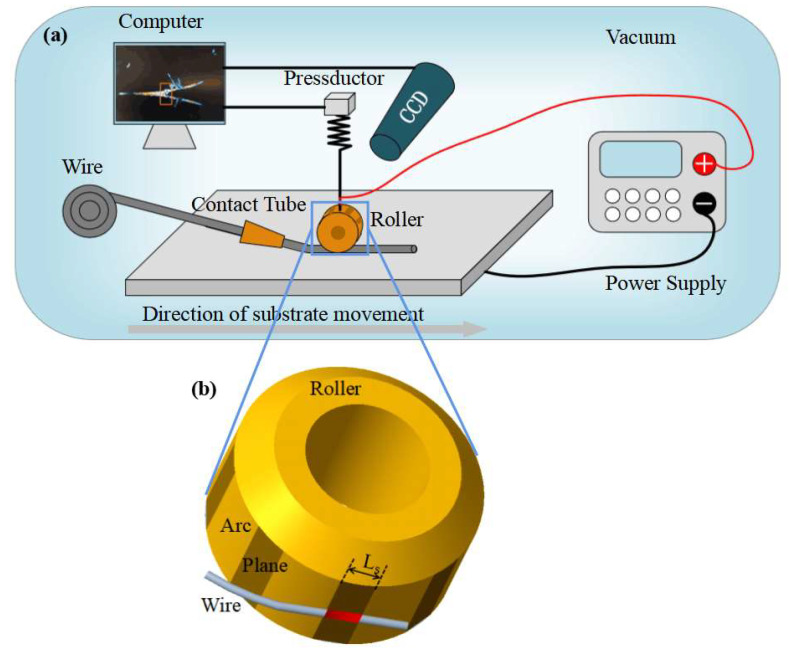
Schematic diagram: (**a**) Schematic of SP-JHAM experimental system, (**b**) are contact length (*L*_s_).

At the same time, *R* includes the resistance and contact resistance of the wire itself:

(2) $$R=R_{1}+R_{2}+R_{3}$$

where *R*_1_ is the resistance of the wire itself, *R*_2_ is the contact resistance between the wire and the roller, and *R*_3_ is the contact resistance between the wire and the substrate. Feulvarch et al. [32] found that the contact resistance of low-carbon steel is closely related to pressure, whose relationship is shown in Formula (3).

(3) $$R_{c}\left(P\right)=0.1766629\times P^{-0.6983364}$$

where *R*_*c*_(*P*) is the contact resistance of a low-carbon steel plate, and *P* is the contact pressure which is calculated by Formula (4).

(4) $$P=\frac{F}{S}$$

where *F* is the electrode pressure of the copper roller to the wire, and *S* is the contact area between the wire and the copper pressure head. When the roller contacts the wire, the machine tool continues to move down, generating electrode pressure on the contact part of the wire (as shown in the red part of Figure 2b). Many scholars have shown that electrode pressure plays a very important role in resistance spot welding [33,34]. Appropriate pressure can maintain resistance stability and thus affect the generation of appropriate Joule heat, which provided a partial reference for this study. The contact area between the wire and roller varies with the area of roller. The contact length between the wire and roller can be approximately regarded as line contact; the wire diameter remains the same simultaneously. Therefore, the contact area *S* between the wire and roller is controlled by controlling the width *L*_s_ of the roller.

In this study, a SP-JHAM experimental system was built, whose schematic diagram is shown in Figure 2a. According to the scholars’ research and previous experimental experience, the copper roller was specially designed in this study [27,35] (as shown in Figure 2b). To save materials and convenience, multiple planes were milled on one roller (shown in the dark area in Figure 2b). During the printing process, the roller was fastened by nuts and screws and did not rotate, which ensured stable contact between a plane and wire and accomplished sliding forming. Multiple planes were milled on one roller because after a plane was damaged, it was only necessary to lose nuts and screws holding the roller and change one plane rather than a whole roller. The contact between the red part of the wire and the roller is the contact between the cylinder and the plane, while the rest of the wire and the roller are not in contact. The contact area between the wire and the roller can be changed by controlling *L*_s_.

### 2.3. Methods

The surface appearance of the printed part can preliminarily reflect the quality of the printed layer. In this paper, the printed tracks under different process parameters are visually compared and analyzed, and the surface-forming appearance is scored. The main factors dictating the score are whether a single track can be formed and adhered to the substrate, whether the surface is smooth, and whether there are surface defects, etc. The specific scoring standard is as follows: points of 0–2 mean the track is unable to adhere to the substrate or form; points of 3–5 mean a poor single pass forms, or obvious defects; points of 6–7 mean good forming, but uneven or discontinuous printing; and points of 8–10 mean a single good pass and smooth surface forms.

**Figure 3:**
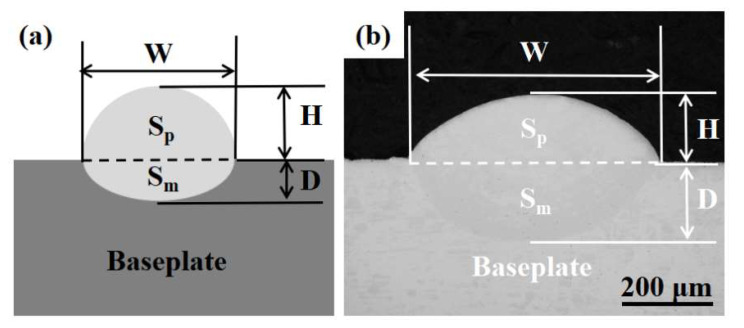
Geometry of the single-layer section: (**a**) schematic, (**b**) actual.

Figure 3 defines the main parameters of a typical single-pass cross-sectional geometry, with the upper part being the melting area of the wire and the lower part being the molten pool. The main measured parameters include melting width (*W*), melting height (*H*), and melting depth (*D*). The aspect ratio and dilution ratio of the cladding layer are two important indexes to evaluate the quality of the printed layer, which can be used to evaluate the three-dimensional stacking ability of the printed layer [36]. The aspect ratio of the printed layer refers to the ratio of the width to the height of the cladding layer, which has an important effect on multi-pass lapping. The larger the aspect ratio of the printed layer, the smaller the lapping area, the better the forming quality of the printed layer, and the stronger the lateral expansion ability will be [37]. If the dilution ratio is too high, the base material components will diffuse too much in the melting layer, which will easily lead to cracking of the melting layer, or defects such as air holes, thereby reducing the performance of the printed parts. The smaller the dilution ratio is, the stronger the vertical expansion ability is. However, if the dilution ratio is too small, the printing layer and the substrate will not be firmly bonded and will be easy to peel off [38]. The aspect ratio and dilution ratio are shown in the specific Formulas (5) and (6),

(5) $$\eta =\frac{D}{D+H}\times 100\%$$

(6) $$\frac{W}{H}=\frac{Width}{Height}$$

where *η* is the dilution rate of a cladding layer, *D* is the depth of a molten pool, *H* is the height of a cladding layer, *W* is the width of a cladding layer, and *W*/*H* is the aspect ratio of a cladding layer.

Through a large number of previous studies by this research group, it was found that the factors affecting the size and morphology of single-layer weld beads manufactured by additive based on the Joule heat source include supply current, electrode pressure, contact area, dry elongation of wire, guide wire angle, voltage, vacuum degree, moving speed of platform [27], etc. In this paper, an experiment was designed regarding the effects of power supply current, electrode pressure, and contact area on the forming appearance, aspect ratio, and dilution rate. When conducting a single-channel fuse test, it was found that the current is from 180–300 A, with each increment of 20 A; the pressure is from 0.4–0.7 N, with each increment of 0.05 N, and the contact length is from 0.5–1.7 mm, with each increment of 0.2 mm.

In order to eliminate the effects of other factors on the experimental results, through the previous research of our research group, the parameters were set as follows: the dry elongation of the wire was 12 mm, the angle between the wire guide head and the substrate was 30°, the upper voltage limit was 4 V, the vacuum degree was 7 × 10^−2^ Pa, and the moving speed of the platform was 150 mm/min [27]. At the same time, considering the effects of interaction on the experimental results, a “three factors and three levels” Taguchi method based on the single-factor experimental results was designed; this determines the primary and secondary order of each factor’s effect and obtains the optimal combination of process parameters.

After CNC milling, the substrate surface was cleaned with absolute alcohol and dried for later use. The surface roughness of the printed parts was detected by a confocal detector Smartproof5 (ZEISS, Oberkochen, Germany), and the internal pores and other defects were analyzed by Vtomex M (PHOENIX, Blomberg, Germany) industrial computerized tomography (CT). A wire-cutting machine is used to cut along the cross-sectional direction of the printed layer and make the sample. The sample is ground by sandpaper with particle sizes of 120, 260, 400, 600, 800, 1000, 1200, 1500, and 2000 and then polished. After the Kalling solution erodes the surface, it is immediately washed with absolute ethanol and dried. The parameters such as width, height, and depth of a cladding channel with an ultra-depth of field microscope were measured by VHX-950F (KEYENCE, Osaka, Japan). An optical microscope AX10 (ZEISS, Oberkochen, Germany) was used to observe the microstructure after corrosion and take photos of the microstructure. The chemical composition of the wire and plate was analyzed by a JSM-IT500LA scanning electron microscope (JEOL, Tokyo, Japan) with EDS energy spectrum analysis. The software for analysis and statistics is OriginPro 8 (OriginLab, Northampton, MA, USA), which is used to plot the data in this paper.

## 3. Results and Discussions

### 3.1. Effects of Process Parameters on Surface Appearance and Cross-Section Geometry

**Figure 4:**
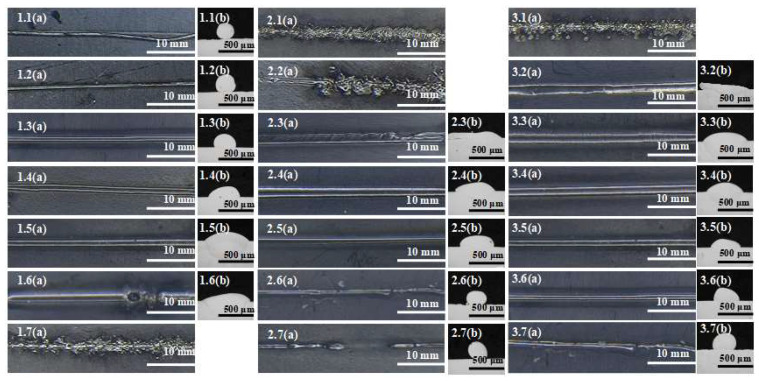
Surface morphology and cross-section geometry of printed layer under different process parameters. 1.1–1.7(**a**) shows the surface morphology of different currents, and 1.1–1.6(**b**) are the corresponding cross-section geometry; 2.1–2.7(**a**) shows the surface morphology of different pressures, and 2.3–2.7(**b**) are the corresponding cross-section geometry; 3.1–3.7(**a**) shows the surface morphology of different contact lengths, and 3.2–3.7(**b**) are the corresponding cross-section geometry.

According to the process parameters and experimental scheme in Table 3, the surface morphology and cross-sectional geometric appearance of the printed layer under various parameters are obtained, as shown in Figure 4.The morphology scoring and measurement data are shown in Table 4.

#### 3.1.1. Effects of Power Supply Current on Surface Appearance and Cross-Section Geometry

**Figure 5:**
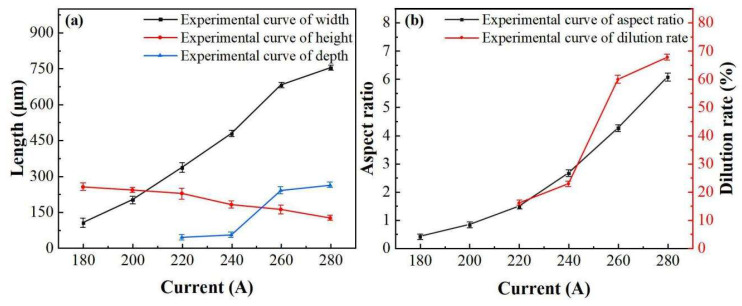
Effect curve of current on cross-section geometry: (**a**) Effects of current on width, height and depth, and (**b**) effects of current on aspect ratio and dilution rate.

The effects of current on the surface appearance are shown in Figure 4, 2.1–2.7. If the current is too low, the wire cannot be completely melted (Figure 4, 1.1) and a typical molten pool cannot be formed, which will cause the wire not to be bonded to the substrate. If the current is too high (Figure 4 1.7), the wire will burst and cannot be formed. As shown in Figure 5, when the current is between 180 A and 220 A, the melting width, height, and width–height ratio change slowly, and the dilution rate is 0%. This is because when the current is low, the Joule heat generated is mainly absorbed by the wire. The wire will become flat with the increase in current, but the substrate temperature is low and the heat dissipation is fast. The heat is insufficient to form a high-temperature molten pool between the wire and the substrate, which is why the dilution rate is 0. With the increase in deposition current, the melting width, depth, aspect ratio and dilution ratio increase, while the melting height decreases. This is because the Joule heat generated at this time is high enough; therefore, the wire can be fully melted and spread out. Simultaneously, the temperature at the contact position with the substrate reaches the melting point of metal, which can then form a deep molten pool (Figure 5).

#### 3.1.2. Effects of Electrode Pressure on Surface Appearance and Cross-Section Geometry

**Figure 6:**
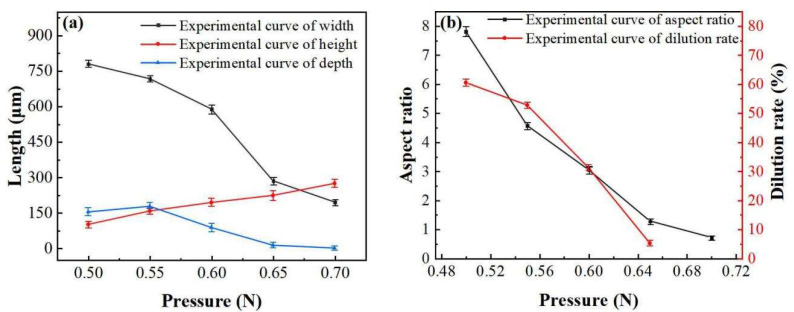
Effects curve of pressure on cross-section geometry: (**a**) Effects of pressure on width, height and depth, and (**b**) effects of pressure on aspect ratio and dilution rate.

The effects of electrode pressure on the surface appearance are shown in Figure 4, 2.1–2.7. If it is too low, the wire will burst (as shown in Figure 4, 2.1 and 2.2), or the layer will be too flat, with a bad appearance (Figure 4, 2.3). When the pressure is too high, the wire cannot be melted and easily broken (as shown in Figure 4, 2.7). According to the Formulas (1), (3) and (4), when the contact area is constant and the pressure f increases, the pressure will increase, the contact resistance *R*_2_ will decrease, and the Joule heat *Q* will decrease with the same other conditions. When the pressure is too small (0.40 N—0.45 N), the high temperature generated by the wire at the moment of electrifying makes the wire fuse and burst, and it cannot be formed. When the pressure is between 0.50 N and 0.70 N, the melting width, depth, aspect ratio, and dilution rate all decrease with the increase in pressure (Figure 6), while the melting height increases. This is because the Joule heat generated by the pressure in this range will not blow up the wire. The heat decreases with the increase in pressure, resulting in the above phenomenon.

#### 3.1.3. Effects of Contact Length on Surface Appearance and Cross-Section Geometry

**Figure 7:**
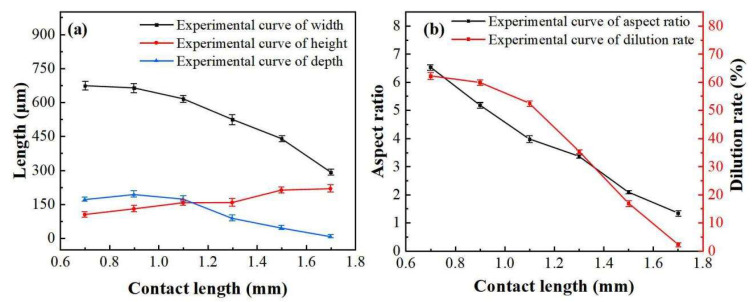
Effects curve of contact length on cross-section geometry: (**a**) Effects of contact length on width, height and depth, and (**b**) effects of contact length on aspect ratio and dilution rate.

The effects of contact length on the surface appearance are shown in Figure 4, 3.1–3.7. If the contact length is too short, the wire will burst (Figure 4 3.1) or spread out excessively and unevenly (Figure 4 3.2). According to the Formula (1), (3) and (4), when the pressure is constant, the contact area will be increased, and the pressure per unit area and the contact resistance *R*_2_ will be reduced. Because the moving speed is consistent, the heating time *t* and the current will be constant, the Joule heat *Q* generated will be reduced. Effects curves of contact length on cross-section geometry are shown in Figure 7; with the increase in *L*_s_, the melting width, melting depth, ratio of width to height, and dilution ratio decrease, while the melting height increases, which is caused by the decrease in heat.

### 3.2. Optimization of Process Parameters

The above experimental results show that there are apparent effects of a single process parameter on surface morphology and cross-section geometry size. However, the interaction between different process parameters is still unclear. In this paper, nine groups with good forming appearance were selected from single-factor experiments. The “three factors and three levels” experiment is designed based on the Taguchi method. The experimental design is shown in Table 5.

The Taguchi method results with the same process parameters are shown in Table 6. The range analysis method is used to analyze the results of orthogonal experiments. The mean value *k*_i_ reflects the trend of various factors affecting the experimental indexes at different experimental levels. The horizontal range *R* of each factor is calculated by the mean value *k*_i_, as shown in Formula (7). The magnitude of the range *R* is used to indicate the importance of experimental factors. Usually, the larger the *R* is, the greater the effects of this factor on the experimental results will be [39]. Finally, the best combination of process parameters is obtained by combining the optimal level of experimental results of various factors.

(7) $$R=k_{i}\;max-k_{i}min$$

where *R* is the extreme horizontal value of each factor, *k*_*i*_ *max* is the maximum value of the mean value *k*_*i*_, and *k*_*i*_ *min* is the minimum value of the mean value *k*_*i*_.

The calculation results of different water mean values ***k**_i_* and range ***R*** are shown in Table 7. The results show that the primary and secondary order of the effects of different process parameters of Joule heat additive manufacturing on the aspect ratio is as follows: pressure, current, and contact length. If only increasing the aspect ratio is considered, the scheme A_3_B_1_C_1_ is preferable. As for the dilution rate, the order of the three factors is pressure, current, and contact length. If only reducing the dilution rate is considered, scheme A_1_B_3_C_3_ can be adopted.

Through actual printing, the appearance of the two groups of experimental surfaces is not ideal. In the former, the current is too large and the pressure and contact length are too small, resulting in a too-low melting height of the cladding layer, an unsmooth surface, and an extremely unstable printing process. The latter’s current is too small, and the pressure and contact length are too large, which will lead to the inability to form a stable molten pool on the substrate. At the same time, the wire cannot be completely melted and cannot be combined with the substrate. The literature [40,41] shows that when the dilution rate is 30~40% and the aspect ratio is greater than 2, the printed layer can form a good metallurgical bond with the substrate, which is conducive to multi-layer construction. Moreover, the obtained specimen has a high fatigue life, that is, the printed layer has good performance. The results indicate that when ***I*** = 260 A, ***F*** = 0.60 N, and ***L***_s_ = 1.3 mm, the printing process is stable, and a good printing layer can be obtained. At this time, the cross-sectional shape parameters are ***W*** = 496 μm, ***H*** = 235 μm, ***D*** = 106 μm, ***W***/***H*** = 2.11, ***η*** = 31.17%.

### 3.3. Characteristic Analysis

**Figure 8:**
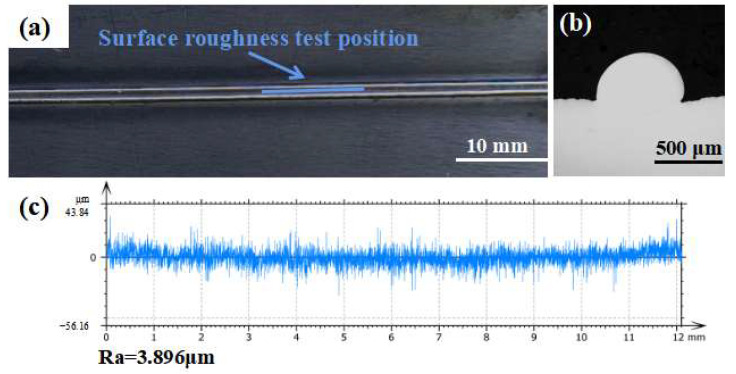
Surface quality: (**a**) surface morphology, (**b**) cross-section geometry, and (**c**) surface roughness along the printing direction.

The surface roughness of 3D printed parts is an important standard to evaluate the surface quality of parts, which determines the surface effect and overall quality of 3D printed parts. Lower roughness results in better wear and corrosion resistance of the parts and greatly reduces the post-processing workload [42,43]. Figure 8a shows the macroscopic appearance under the optimal process parameters, showing that the surface is smooth, the printing is continuous and the width is uniform. The surface roughness is measured and calculated based on ISO4287. Figure 8b displays the surface contour extracted from the layer scanned by the confocal microscope along the printing direction, with an average surface roughness (Ra) of 3.896 μm, which is better than that of WAAM: 200 μm [44], EBAM: 15 μm [45], and LMD: 8 μm [46].

**Figure 9:**
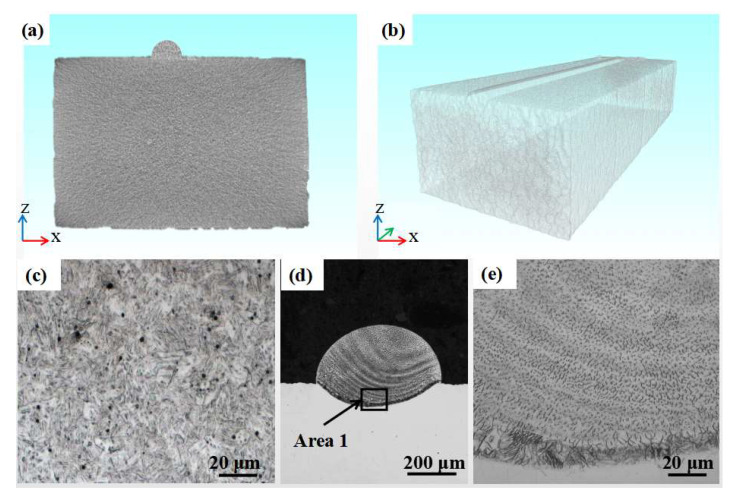
Internal quality: (**a**) CT scan image at depth of 12 mm, (**b**) transparent view obtained from CT scan analysis of printing layer, (**c**) micro-morphology of original wire, (**d**) micro-morphology of printing layer cross-section, and (**e**) enlarged view of Area 1.

In the process of additive manufacturing, the wire melts, precipitates, and solidifies, thereby realizing metallurgy; the whole process is completed in a very short time, and the formed parts are prone to defects, such as holes, cracks, spheroidization and splash, which seriously affect the quality and performance of printed parts. Through industrial CT, the internal defects of objects can be nondestructively detected, and defects such as holes and cracks of parts can be visually and accurately observed [47,48,49,50]. Figure 9a,b show CT scanning results. The industrial CT test of the printed samples found that there are no typical defects such as holes and cracks (it should be pointed out that if there are holes and cracks, the results will be displayed in bright colors). Figure 9d,e show the micro-morphology of the printing layer cross-section, demonstrating that the wire and the substrate are completely metallurgically bonded.

## 4. Conclusions

In this study, the process characteristics of 304 stainless steel wire printing are investigated using the self-developed experimental system of SP-JHAM. The specific conclusions are as follows:

1. In a given range, with the increase in current, melting height decreases; some indexes in this work, such as melting width, melting depth, aspect ratio, and dilution rate, increase. With the rise in pressure and contact length, the melting width, melting depth, aspect ratio, and dilution rate all decrease, while the melting height increases.
2. Electrode pressure, power supply current and contact length significantly affect the forming process, but the electrode pressure has the greatest effect on the aspect ratio and dilution rate, followed by the power supply current and contact length.
3. The optimal process parameters are ***I*** = 260 A, ***F*** = 0.60 N, ***L***_s_ = 1.3 mm. With these process parameters, the layer surface is smooth, printing is continuous and width is uniform. Surface roughness Ra is 3.896 μm, the aspect ratio is 2.11, the dilution rate is 31.17%, and the printed layer forms a good metallurgical bond with the substrate. In addition, there are no obvious cracks, pores and other defects inside.
4. This additive manufacturing method is still experimental. Although the single-layer stable forming of 304 stainless steel wire can be accomplished, the control system and mechanical structure need to be further optimized to accomplish the stable forming of multi-layer and complex shapes. The printing efficiency needs to be further improved for large metal parts, and the forming process of titanium alloy, aluminum alloy, and other metal materials needs further study. In the future, this method could also be applied to additive manufacturing in outer space.

## Figures and Tables

**Table 1:** Chemical compositions of 316 L and 304.

Material	Elements	Cr	Mn	Mo	Ni	Si	C	P	S	Fe
316L	Wt%	16.38	0.86	2.11	10.11	0.44	0.018	0.026	0.007	68.33
304	Wt%	18.01	0.77	0.03	8.03	0.47	0.052	0.027	0.003	69.34

**Table 2:** Physical properties of 316 L and 304.

Material	Density (g/cm^3^)	Melting Temperature (°C)	Conductivity (W/m·°C)	Resistivity (Ω·mm^2^/m)
316L	7.98	1400	13.31	0.74
304	7.93	1400	14.63	0.73

**Table 3:** Single-factor experimental parameters.

Parameters	Current (A)	Pressure (N)	Contact Length (mm)
1	[180–300]	0.5	1.1
2	220	[0.4–0.7]	1.1
3	220	0.5	[0.5–1.7]

**Table 4:** Experimental results under different process parameters.

Parameters	Current (A)	Pressure (N)	Contact Length (mm)	Morphology Score	Width (μm)	Height (μm)	Depth (μm)	Aspect Ratio	Dilution Rate (%)
1.1	180	0.55	1.1	2	105	254	0	0.41	0
1.2	200	0.55	1.1	5	201	241	0	0.83	0
1.3	220	0.55	1.1	8	336	227	43	1.48	15.93
1.4	240	0.55	1.1	9	478	180	53	2.66	22.75
1.5	260	0.55	1.1	10	681	160	239	4.26	59.90
1.6	280	0.55	1.1	6	753	124	261	6.07	67.78
1.7	300	0.55	1.1	0	0	0	0	0	0
2.1	260	0.40	1.1	0	0	0	0	0	0
2.2	260	0.45	1.1	1	0	0	0	0	0
2.3	260	0.50	1.1	4	780	100	153	7.80	60.47
2.4	260	0.55	1.1	10	717	157	177	4.57	52.99
2.5	260	0.60	1.1	10	589	193	87	3.05	31.07
2.6	260	0.65	1.1	7	285	223	12	1.28	5.11
2.7	260	0.70	1.1	1	195	274	0	0.71	0
3.1	260	0.55	0.5	0	0	0	0	0	0
3.2	260	0.55	0.7	6	673	103	169	6.53	62.13
3.3	260	0.55	0.9	9	663	128	191	5.18	59.87
3.4	260	0.55	1.1	8	615	155	171	3.97	52.45
3.5	260	0.55	1.3	9	524	156	86	3.36	35.54
3.6	260	0.55	1.5	8	439	211	43	2.08	16.93
3.7	260	0.55	1.7	4	291	217	5	1.34	2.25

**Table 5:** Levels of factors for the Taguchi method.

Factors	Notation	Unit	Level		
			1	2	3
Current	A	A	240	260	280
Pressure	B	N	0.50	0.55	0.60
Contact length	C	mm	1.2	1.3	1.5

**Table 6:** Results of the Taguchi method.

Number	Current A (A)	Pressure B (N)	Contact Length C (mm)	Aspect Ratio *W/H*	Dilution Rate *η* (%)
1	240	0.50	1.1	3.84	42.61
2	240	0.55	1.3	2.31	21.32
3	240	0.60	1.5	0.92	0.00
4	260	0.50	1.3	4.17	51.14
5	260	0.55	1.5	3.06	33.19
6	260	0.60	1.1	2.85	31.76
7	280	0.50	1.5	4.16	43.54
8	280	0.55	1.1	4.63	45.22
9	280	0.60	1.3	3.08	33.97

**Table 7:** Calculation results of the Taguchi method.

Evaluation Indicators	Items	Current A (A)	Pressure B (N)	Contact Length C (mm)
Aspect ratio ***W/H***	***k***_1_	2.357	4.057	3.773
	***k***_2_	3.360	3.133	3.187
	***k***_3_	3.957	2.283	2.713
	***R***	1.600	1.773	1.060
Dilution rate ***η*** (%)	***k***_1_	21.31	45.76	39.86
	***k***_2_	38.70	33.24	35.48
	***k***_3_	40.91	21.91	25.58
	***R***	19.60	23.85	14.29

## Data Availability

The data presented in this study are available upon reasonable request from the corresponding author.
